# BiDFDC-Net: a dense connection network based on bi-directional feedback for skin image segmentation

**DOI:** 10.3389/fphys.2023.1173108

**Published:** 2023-06-20

**Authors:** Jinyun Jiang, Zitong Sun, Qile Zhang, Kun Lan, Xiaoliang Jiang, Jun Wu

**Affiliations:** ^1^ College of Mechanical Engineering, Quzhou University, Quzhou, China; ^2^ Department of Rehabilitation, The Quzhou Affiliated Hospital of Wenzhou Medical University, Quzhou People’s Hospital, Quzhou, China

**Keywords:** image segmentation, skin, bi-directional feedback, dense connection, U-Net

## Abstract

Accurate segmentation of skin lesions in dermoscopic images plays an important role in improving the survival rate of patients. However, due to the blurred boundaries of pigment regions, the diversity of lesion features, and the mutations and metastases of diseased cells, the effectiveness and robustness of skin image segmentation algorithms are still a challenging subject. For this reason, we proposed a bi-directional feedback dense connection network framework (called BiDFDC-Net), which can perform skin lesions accurately. Firstly, under the framework of U-Net, we integrated the edge modules into each layer of the encoder which can solve the problem of gradient vanishing and network information loss caused by network deepening. Then, each layer of our model takes input from the previous layer and passes its feature map to the densely connected network of subsequent layers to achieve information interaction and enhance feature propagation and reuse. Finally, in the decoder stage, a two-branch module was used to feed the dense feedback branch and the ordinary feedback branch back to the same layer of coding, to realize the fusion of multi-scale features and multi-level context information. By testing on the two datasets of ISIC-2018 and PH2, the accuracy on the two datasets was given by 93.51% and 94.58%, respectively.

## 1 Introduction

**FIGURE 1:**
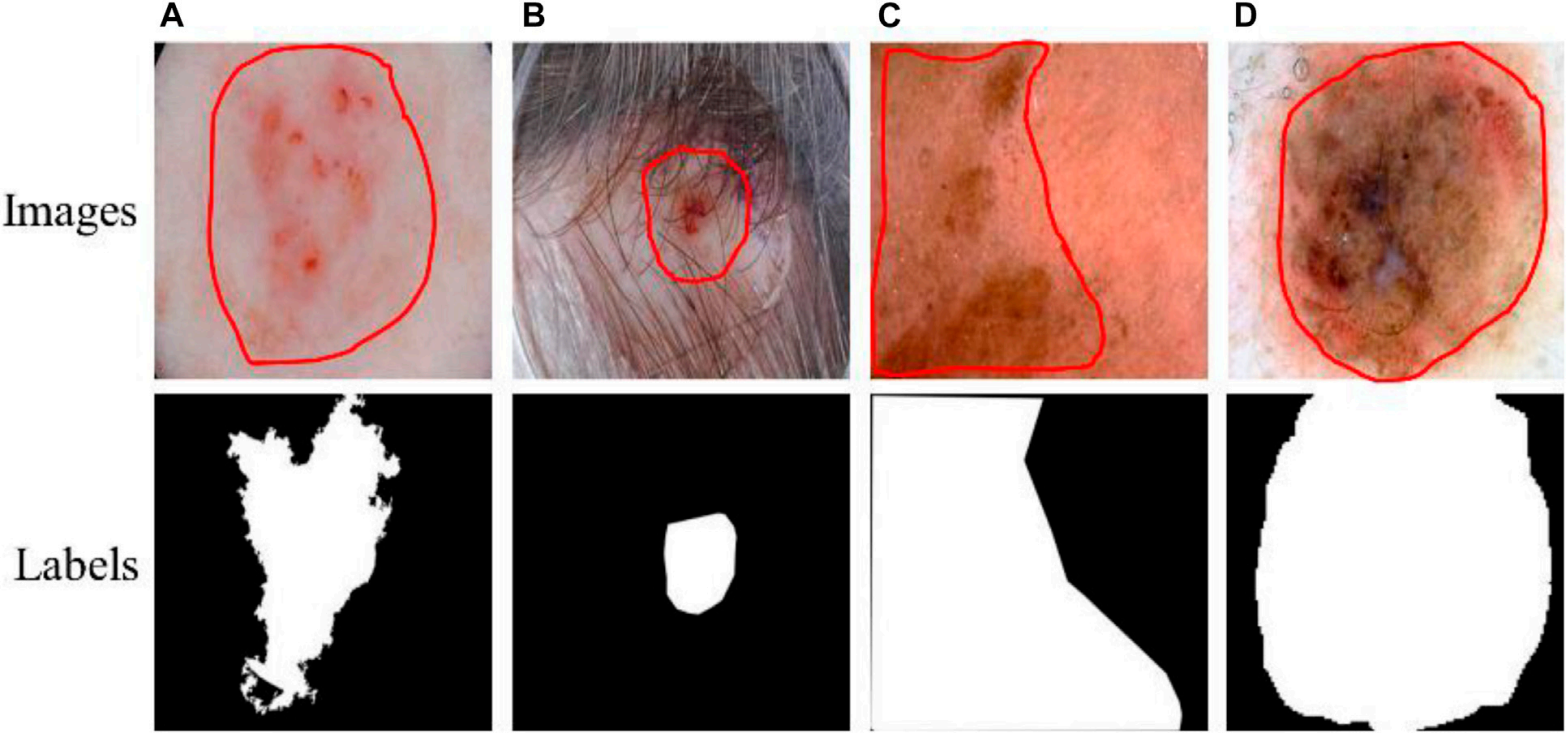
Lesions and corresponding labels. **(A)** Irregular and fuzzy contours, **(B)** Hair, **(C)** Ebony, **(D)** Bubbles.

Skin cancer is an extensive, invasive, and fatal cancer. In the early stage, the characteristics of the lesions are not obvious, and very similar to other skin diseases, resulting in rapid growth and spread of cancer cells. Therefore, the early detection of lesions is very important to save the lives of patients. At present, skin diagnosis is mainly based on the use of dermatoscopic imaging technology to visualize the diseased area, which can improve the doctor’s visual clarity and relieve visual fatigue. However, the number of physicians with clinical experience is small, and the diagnostic decisions of this experience may be inaccurate or subjective. Recently, with the development of computer vision, computer-aided diagnosis technology has been widely used in the field of medical image segmentation. Due to the irregular and fuzzy contours of some lesions and the presence of interference information such as hair, ebony, and bubbles in the images, it is difficult to achieve accurate segmentation, as shown in Figure 1. Therefore, it is of great value to study an efficient and accurate segmentation method in both medical and academic fields.

In the past, researchers have published many segmentation algorithms for skin images. Generally, these methods can be classified as traditional learning and deep learning. For the traditional segmentation methods, there was a genetic algorithm (Rouhollahi et al., 2020; Da Silva et al., 2021), a multilevel threshold method (Resma and Nair, 2021; Singh et al., 2021; Abualigah et al., 2022), watershed (Genitha et al., 2020; Indriyan et al., 2021), fuzzy clustering (Li, 2020; Qu and Wang, 2021), partial differential equation (Guo et al., 2020; Tian and Chen, 2020) and so on. These methods utilized digital image knowledge and mathematical equations to define the segmentation features, so they have the characteristics of simple calculation and fast segmentation speed. Nevertheless, they also have some disadvantages. For instance, the watershed algorithm may iteratively label gradient images for image segmentation. However, subtle changes such as the noise of the image and the texture of the object may affect the smoothness of the image gradient, causing it to over-segmentation. In multi-level thresholds, the optimum set of thresholds can divide the image into several regions. But it is difficult to choose a suitable range. Because not only does it rely heavily on a large number of controlled parameter values, but it is also expensive and complicated to operate. Its segmentation effect is also not ideal, especially for images with different kinds of noise or low contrast between foreground and background. In a word, they are sensitive to image noise and easily cause low segmentation accuracy. Moreover, they need to define a large number of parameters manually and extract the characteristics which leads to a low ability to generalize.

With the advent of computational techniques and artificial intelligence, deep learning starts to be applied to medical images. Its most prominent feature is that it has a convolutional neural network (CNN) (Tyagi and Mehra, 2020), which is similar to brain neurons. Not only can it learn the features of some local regions, but it is also sensitive to the size, position, and orientation of objects. As there is a loss of image detail in convolution and pooling, it is not possible to segment the specific contours of an image exactly. For this reason (Shelhamer et al., 2017), proposed a full convolutional network to effectively achieve image segmentation by replacing the convolutional layer with the fully connected layer. However, image segmentation only depends on the context information, so the segmentation results are not precise enough. Hence, some researchers have proposed model frameworks for variations of FCN, such as SegNet (Badrinarayanan et al., 2017), RefineNet (Lin et al., 2017), and U-Net (Ronneberger et al., 2015). Among them, Ronneberger et al. proposed that the U-Net network structure achieved a better segmentation effect in cell segmentation and others, which attracted many scholars to participate in the improvement of its model. Badshah et al. (Badshah and Ahmad, 2021) fused the residual block and the Bi-directional BConvLSTM network in the U-Net neural network, called ResBCU-Net. First, it uses residual blocks and batch normalization in the encoding path to prevent learning too many extraneous features and avoid overfitting. Then, dense convolutional connections are used in the bottleneck part of the model to enhance feature propagation and encourage feature reuse. Finally, a bidirectional long-short-term memory network is added to the decoding path to avoid gradient vanishing during long-term training. Yang et al. (Yang et al., 2020) proposed that the multiscale U-Net network uses multi-scale spatial pyramid pooling at the end of the down-sampling path to expand the feature receptive field, and uses the expanded convolutional residual block to improve the skip connection, which has good performance in brain tumor segmentation. Du et al. (Du et al., 2021) proposed a densely connected U-Net structure with multi-scale features. The idea of the model is mainly to use the initial block structure and the dense block structure of U-Net to achieve multiple feature extraction and efficient utilization through a string or parallel connections.

The above methods are suitable for the automatic segmentation of most skin images. However, its accuracy and robustness are still insufficient due to irregular object segmentation and imbalance between target and background, which severely hinders the application of deep learning methods in medical diagnosis. To overcome the shortcomings of traditional U-Net in the automatic segmentation of skin images, this is precisely what we focus on in this paper. Here, we proposed a novel bidirectional feedback dense connected network framework called BiDFDC-Net. The main contributions of this paper are as follows.

(1) After convolving the encoder in U-Net, the edge module was introduced to eliminate the problem of gradient vanishing and extract richer multiscale feature information.
(2) The dense connection network was applied to strengthen information fusion between U-Net layers. Each layer reads the state from its previous layer and writes it to the next layer, helping to train a deeper network architecture. In addition, dense connections have a regularizing effect, reducing overfitting to the training set.
(3) Using the two-branch module of dense feedback branch and ordinary feedback branch instead of single-branch upsampling can ensure that the network still has high segmentation accuracy in the case of deepening.
(4) We tested the proposed BiDFDC-Net network and compared it with some classical models and U-Net networks on the ISIC-2018 dataset. Experimental results showed that the BiDFDC-Net network achieves remarkable performance. In addition, to verify the accuracy and appropriateness of the network, the network trained on the ISIC-2018 dataset was tested on a common dataset named PH2.

## 2 Materials and methods

**FIGURE 2:**
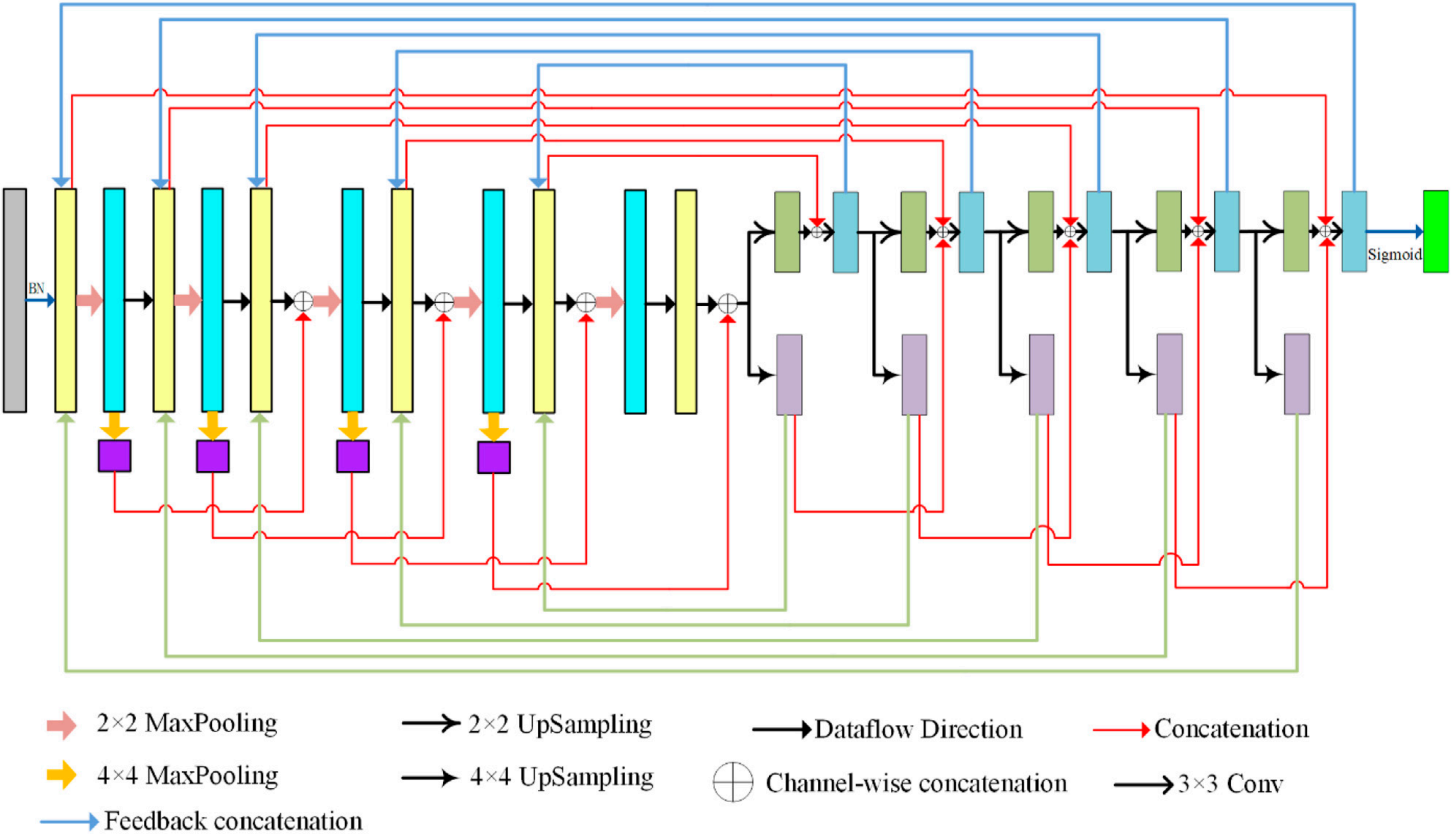
Proposed BiDFDC-Net architecture.

In this section, we present the details of our model for proposing a bi-directional feedback dense connection network framework (called BiDFDC-Net). The network framework is shown in Figure 2. The network model mainly fused shallow features and deep feature semantic information through dense connection, which reduces the loss of effective features caused by operations such as convolution to a certain extent. At the same time, the edge module structure was used as the main component of the coding structure to extract the relationship between different pixels in the image, and further extract the feature information of the image. Finally, the output results of each layer under each path of the decoded part were fed back to the corresponding layer in the encoding network to enable feedback connections and thus enhance the reuse of features and information transfer. And that will make the effect of sample training more efficient and flexible. We will introduce our bi-directional feedback, edge modules, and dense connections in the following subsections.

### 2.1 Bi-directional feedback

**FIGURE 3:**
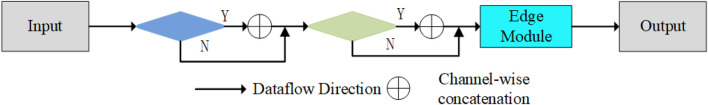
The BiD feedback of the BiDFDC-Net.

The mapping between the input and output of the network model in the network learning process uses context-dependent information. However, their ability to obtain contextual information is limited, and the network recursion will make the network segmentation ability gradually decline. To this end, we feed the decoding information back to the encoding in both directions (BiD), to obtain more global information and retain more effective features. In the model, we feed back the output under each path of the two-branch decoding path to the coding layer. Their feedback connections are shown in Figure 3. It will judge whether there is feedback information at each connection. It is connected if there is information input, otherwise, it is skipped. That makes the image back to the original size, the accuracy has improved. In addition, similar to U-Net, skip connections are used in our network to fuse the semantic features of the encoding and decoding parts, as shown in Figure 2.

### 2.2 Edge module

**FIGURE 4:**
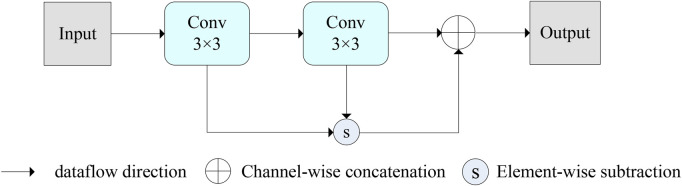
The edge module of the BiDFDC-Net.

To improve the accuracy of image segmentation, an edge module was created in the encoder to extract the edge information of the image. The structure of the edge module is illustrated in Figure 4. The working principle is to use the traditional edge detection technique, which is also the principle of Gaussian difference. It was integrated into the convolution to capture the edge information of the image and reduce the influence of other noise points on the segmentation effect. Its construction is mainly composed of two identical 3 × 3 convolutional layers, element-by-element subtraction, and joining. The purpose of convolutional layers is to obtain the feature information of the image. Other operations are to preserve image edge features at different semantic scales. It is similar to a Gaussian filter, which is equivalent to adding a filter in the image to remove the effect of other irrelevant backgrounds such as irrelevant noise or artifacts on the image segmentation. Its purpose is to extract the edge information of the image and hence improve the sensitivity of the image boundaries. Its formula is expressed as:

$$Output_{Edge}=\left(Conv\left(input\right)-Conv\left(Conv\left(input\right)\right)\right)⊕Conv\left(Conv\left(input\right)\right)$$ (1)

Where *Output*_Edge_ is the Output of the edge module, Conv represents the convolution operation, *input* is the input of the module.

### 2.3 Dense connections

**FIGURE 5:**
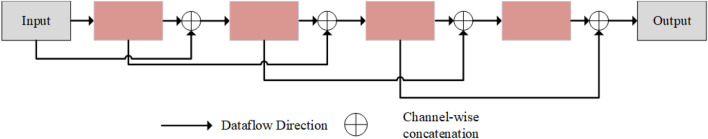
The structure of dense connection in this study.

As displayed in Figure 5, we designed a dense connection to form a certain connection between the layers of the network, to integrate the semantic information and characteristic information between different layers. In contrast to other dense concatenations, we did not take the input of the connection of the local layer from the output of all previous layers but only utilized the parallel output results of the previous layers for concatenation. The connected input in the coding network is the result of parallel underground sampling. In decoding, the concatenation operation is performed by parallel up-sampling results. This not only reduces the learned feature parameters but also improves the network training speed. Moreover, this makes the network model more flexible and easier to migrate. In decoding, we turned the original single path into a double path up-sampling. A path is simply an up-sampling operation that passes information to the next level. The other path operation is to connect the up-sample results with the input information of the upper layer, and then carry out two 3 × 3 convolutions. This will allow the network to add more detail, resulting in a more accurate output.

## 3 Experiments and results

The dermoscopic images from the ISIC-2018 (Tschandl et al., 2018) and PH2 (Garg and Balkrishan, 2022) datasets were utilized to assess the BiDFDC-performance. All models were trained using the Keras API and Tensorflow as the backend in Python 3. The experimental environment is built around a workstation running Windows 10 and equipped with a 24 GB NVIDIA Quadro RTX 6000 GPU. The segmentation network is trained using the Adam optimizer (learning rate = 0.001) (Cui et al., 2023) with a batch size of 8 and an epoch of 300. Every epoch, the validation loss, which was calculated using the Dice loss (Ma et al., 2021), was monitored, and the best model was kept when the validation loss was the smallest during training.

### 3.1 Evaluation metrics

Several quantitative metrics, including Sensitivity (SN), Specificity (SP), Accuracy (ACC), Dice index coefficient (DIC), and Jaccard score (JAC), were applied to evaluate the performance of the models. The following is a diagram of the mathematical formula for each of these metrics:

$$SN=\frac{TP}{TP+FN}$$ (2)

$$SP=\frac{TN}{TN+FP}$$ (3)

$$ACC=\frac{TP+TN}{TP+TN+FP+FN}$$ (4)

$$DIC=\frac{2TP}{2TP+FN+FP}$$ (5)

$$JAC=\frac{TP}{TP+FN+FP}$$ (6)

where TP, TN, FP, and FN represent the number of true positives, true negatives, false positives, and false negatives at the pixel level, respectively.

### 3.2 Experimental results on two datasets

**FIGURE 6:**
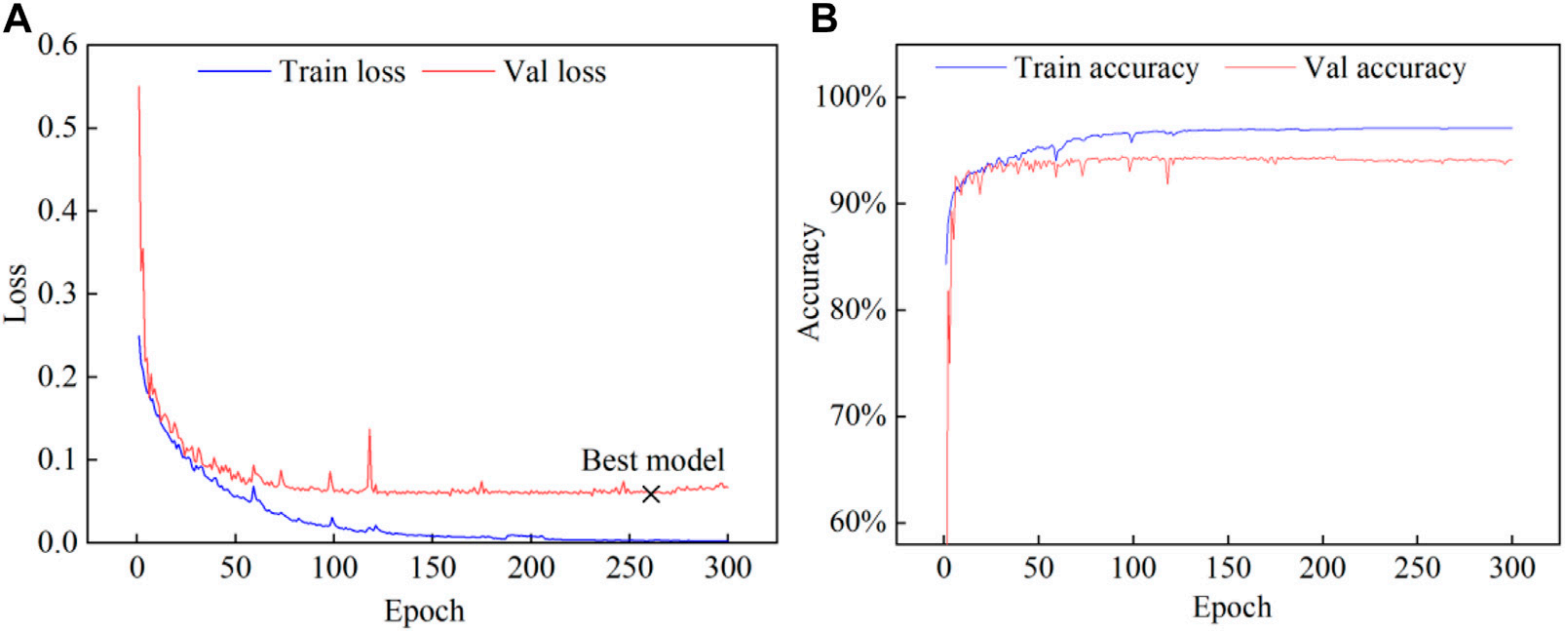
The process of model training on the ISIC-2018 dataset. **(A)** Loss, **(B)** Accuracy.

The change of loss *versus* accuracy during training on the ISIC-2018 dataset is shown in Figure 6. The loss steadily stabilized after 150 epochs, and at epoch 259 the ideal model was attained. The outcomes of the experiment demonstrated that the epochs chosen were appropriate.

**FIGURE 7:**
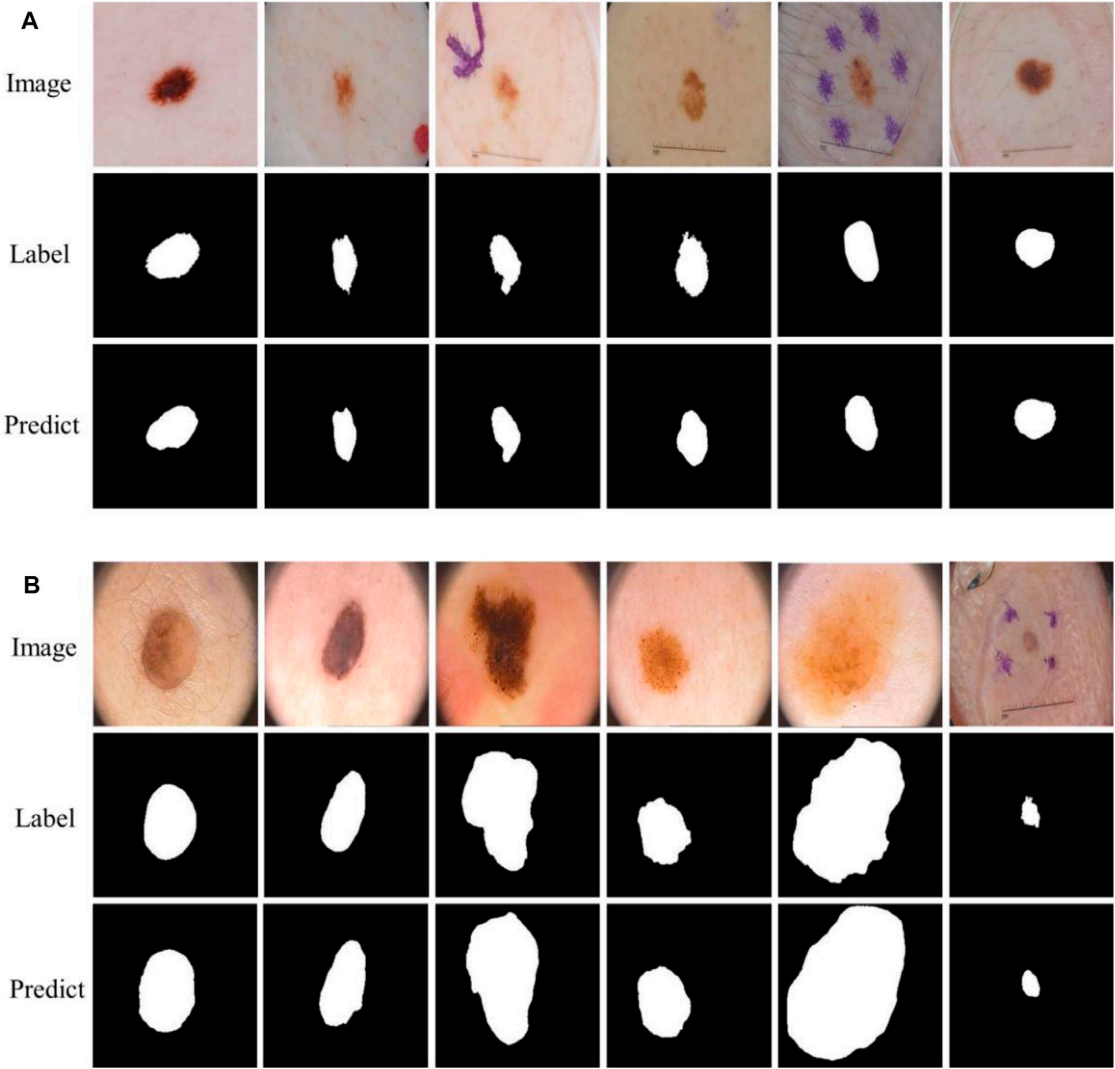
The example of segmentation result. **(A)** ISIC-2018 dataset, **(B)** PH2 dataset.

The evaluation metrics of the model test results on the two datasets are shown in Table 1. Additionally, the example of the segmentation effect of the model on the datasets is shown in Figure 7, which showed that the proposed model can effectively segment abnormal regions and accurately describe the boundaries of the lesion area from dermoscopic images.

**TABLE 1:** The results of BiDFDC-Net on two datasets.

Datasets	SN (%)	SP (%)	ACC (%)	DIC (%)	JAC (%)
ISIC-2018	87.55	95.94	93.51	86.42	76.38
PH2	88.78	96.62	94.58	87.88	78.84

### 3.3 Ablation experiment

Ablation experiments were conducted based on the different structures to show how the enhanced structure affected the model. As seen in Table 2, the strategy we proposed produced the best metrics when compared to other methods.

**TABLE 2:** Experiments on datasets with BiDFDC-Net with different structures.

Datasets	Method	SN (%)	SP (%)	ACC (%)	DIC (%)	JAC (%)
ISIC-2018	Base (UNet)	86.11	94.29	92.81	82.37	70.49
	Base + BiD	87.48	94.94	93.50	84.32	73.36
	Base + BiD + Dense	85.51	95.12	93.32	85.00	74.25
	Base + BiD + EE	87.35	94.40	93.40	84.39	73.33
	BiDFDC-Net	87.55	95.94	93.51	86.42	76.38
PH2	Base (UNet)	88.02	90.05	88.12	82.61	70.67
	Base + BiD	86.45	95.08	93.77	83.33	72.08
	Base + BiD + Dense	86.45	96.56	94.26	86.05	76.07
	Base + BiD + EE	87.96	94.77	94.12	84.12	73.18
	BiDFDC-Net	88.78	96.62	94.58	87.88	78.84

**FIGURE 8:**
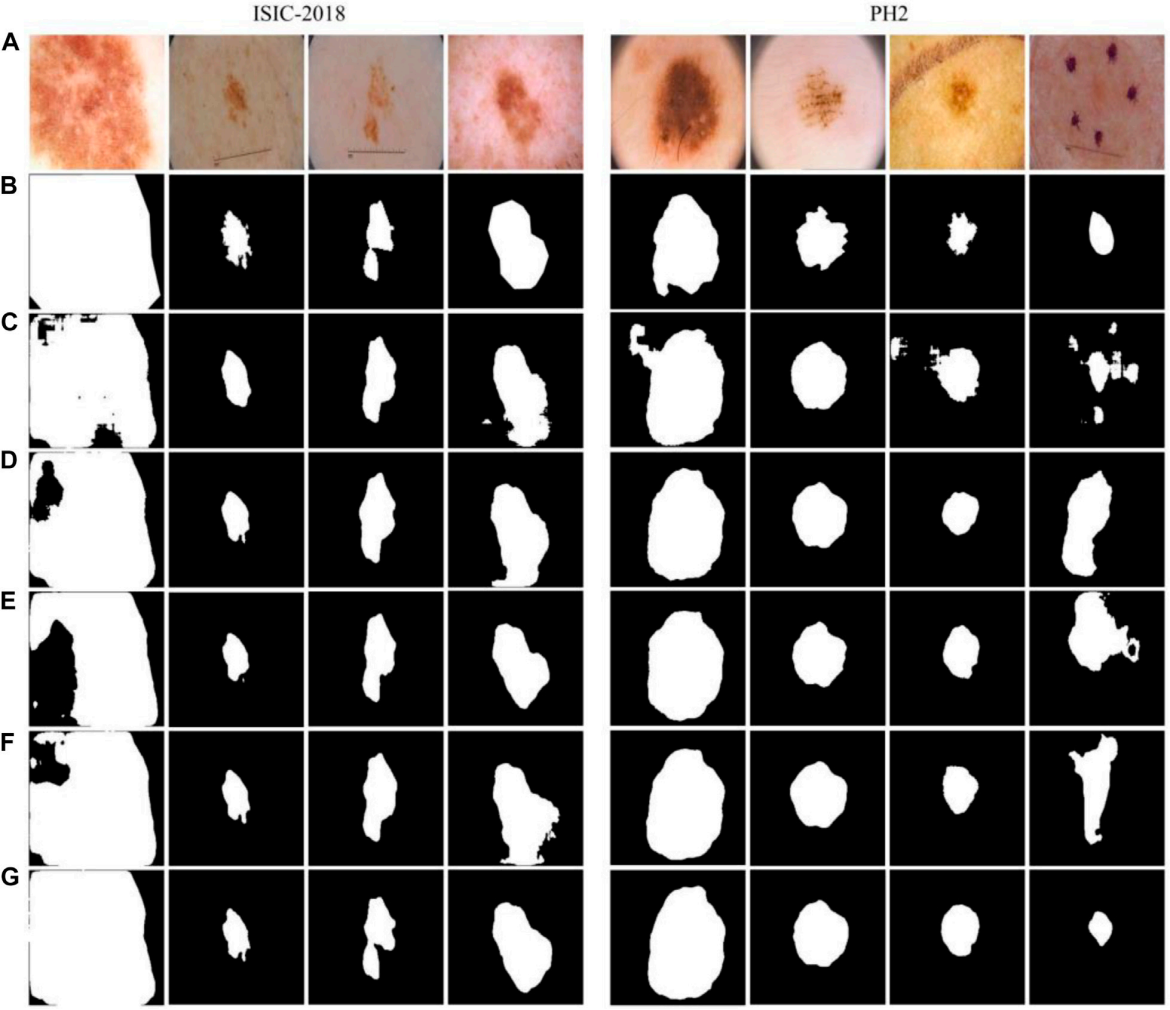
Examples of segmentation results of different structures. **(A)** Images, **(B)** Labels, **(C)** Base, **(D)** Base + BiD, **(E)** Base + BiD + Dense, **(F)** Base + BiD + EE, **(G)** BiDFDC-Net.

Some examples of segmentation were presented to show the impact of each model. Comparing the results in Figures 8C, D, it can be found that the network structure with a bidirectional feedback mechanism classified pixels more stably, and misjudgments of distant pixels were rare. As shown in Figures 8D,E, the model with the Dense connects strengthens and effectively uses the retrieved features but results in information hybrids due to excessive fusion. The network using the Edge module would incorrectly categorize dermoscopic images with significant background interference because there is no semantic relationship between the upper and bottom parts of the image, as seen in Figure 8F. The technique we suggested combines the benefits of both to produce better results, attaining the closest segmentation boundaries to label in the comparison of methods, as shown in Figure 8G.

### 3.4 Comparison with other models

#### 3.4.1 Results of the ISIC-2018 dataset

To illustrate the effect of the proposed method, the model results were compared with some classical models. According to the data in Table 3, BiDFDC-Net outperforms all other models in the comparison in terms of segmentation evaluation metrics.

**TABLE 3:** Comparison of results of different models on the ISIC-2018 dataset.

Method	SN (%)	SP (%)	ACC (%)	DIC (%)	JAC (%)
UNet Ronneberger et al. (2015)	86.11	94.29	92.81	82.37	70.49
SegNet Badrinarayanan et al. (2017)	87.53	93.45	92.48	82.71	70.94
SEUNet Roy et al. (2018)	86.49	95.46	93.46	86.17	76.02
R2UNet Alom et al. (2018)	87.34	95.74	93.15	85.69	75.35
EddyNet Lguensat et al. (2018)	72.78	95.71	90.60	75.82	61.65
HRNet Sun et al. (2019)	87.36	95.59	93.42	85.92	75.77
RS-Net Jia et al. (2022)	86.92	94.27	92.99	83.43	71.85
Ours	87.55	95.94	93.51	86.42	76.38

**FIGURE 9:**
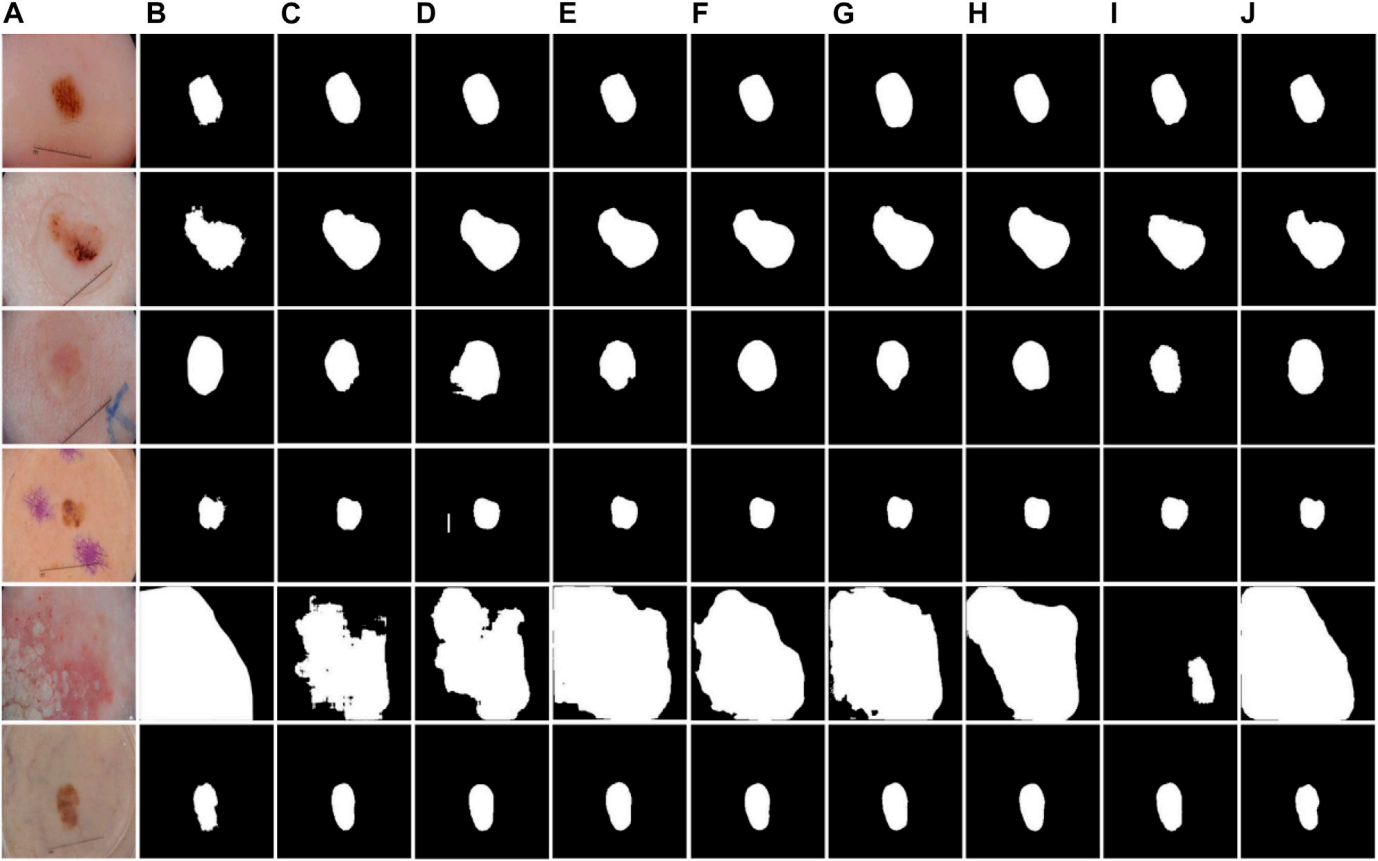
Examples of segmentation results of different models on the ISIC-2018 dataset. **(A)** Images, **(B)** Labels, **(C)** UNet, **(D)** SegNet, **(E)** HRNet, **(F)** RS-Net, **(G)** SEUNet, **(H)** R2UNet, **(I)** EddyNet, **(J)** Ours.

As shown in Figure 9, examples of segmentation results of different models are displayed. Due to the lack of association of high-level and low-level semantic features, UNet, SegNet, R2UNet, and EddyNet will produce missing pixels in the face of some dermoscopic images with large background interference and complex color changes, as shown in Figures 9C, D, H, I. As demonstrated in Figure 9E. Although HRNet fuses the context information to some extent, the lack of the Edge module makes it impossible to describe the edge more finely. For some models with an attention mechanism, although the location of the lesion area is accurate, the delineation of the edge is still lacking, which can be seen in Figures 9F, G. Note that the optimal results are obtained by our proposed method.

#### 3.4.2 Results of the PH2 dataset

**FIGURE 10:**
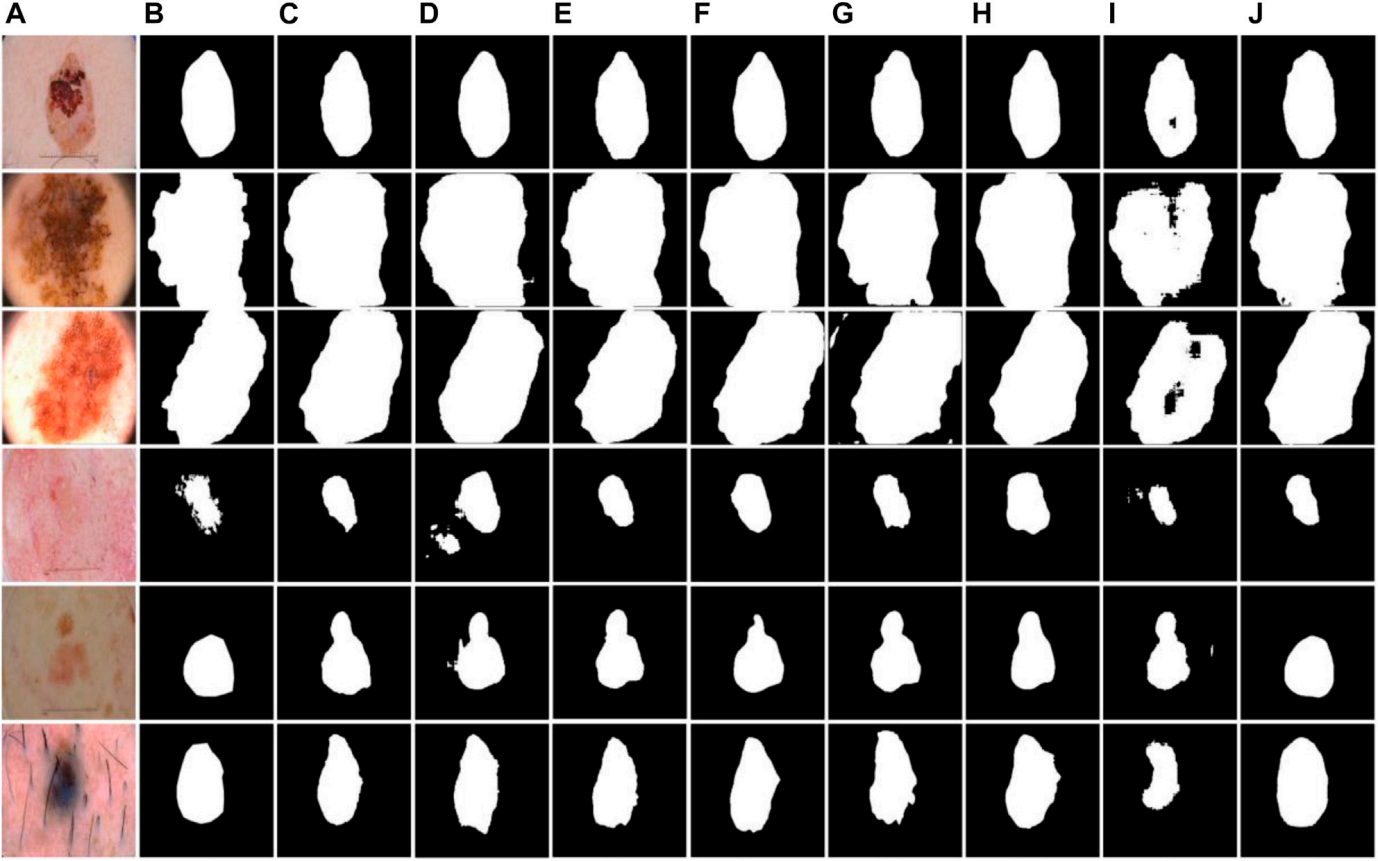
Examples of segmentation results of different models on the PH2 dataset. **(A)** Images, **(B)** Labels, **(C)** UNet, **(D)** SegNet, **(E)** HRNet, **(F)** RS-Net, **(G)** SEUNet, **(H)** R2UNet, **(I)** EddyNet, **(J)** Ours.

The test results of BiDFDC-Net on the PH2 dataset are superior to those of the other models taking part in the comparison in all metrics, as shown in Table 4. Additionally, as shown in Figure 10, the test results of models other than the suggested method on the PH2 dataset would contain mistakes and omissions, which is consistent with the prediction on the ISIC-2018 dataset. The outcomes show that the segmentation performance of the model we suggested on the PH2 dataset also has an excellent performance.

**TABLE 4:** Comparison of results of different models on the PH2 dataset.

Method	SN (%)	SP (%)	ACC (%)	DIC (%)	JAC (%)
UNet Ronneberger et al. (2015)	88.02	90.05	88.12	82.61	70.67
SegNet Badrinarayanan et al. (2017)	86.05	92.36	91.25	79.95	67.67
SEUNet Roy et al. (2018)	87.53	94.18	92.51	84.34	73.58
R2UNet Alom et al. (2018)	88.07	95.13	92.87	86.34	76.50
EddyNet Lguensat et al. (2018)	63.76	94.15	87.12	67.20	52.39
HRNet Sun et al. (2019)	87.38	94.40	92.44	84.98	74.71
RS-Net Jia et al. (2022)	87.83	93.92	92.70	83.59	72.52
Ours	88.78	96.62	94.58	87.88	78.84

As shown in Table 5, we present a comprehensive presentation of the effects of different models. The results show that our network was higher than other models in parameter size, FLOPs, and its processing efficiency was slightly slower than the other models, but these values were still at a relatively respectable size. In addition, for medical images, we paid more attention to the accuracy of recognition, and improving the recognition accuracy at the expense of certain recognition efficiency is desirable for this research. Of course, we will continue to explore segmentation models with higher accuracy and efficiency in subsequent research.

**TABLE 5:** Comparison of performance of different models.

Method	Parameters (M)	FLOPs (G)	FPS
UNet Ronneberger et al. (2015)	1.12	3.74	174.05
SegNet Badrinarayanan et al. (2017)	2.80	20.10	145.29
SEUNet Roy et al. (2018)	1.87	6.93	156.54
R2UNet Alom et al. (2018)	16.83	54.70	72.88
EddyNet Lguensat et al. (2018)	0.04	1.76	180.22
HRNet Sun et al. (2019)	27.28	22.00	78.71
RS-Net Jia et al. (2022)	30.35	35.32	95.88
Ours	65.01	117.47	35.61

## 4 Conclusion

In this study, we proposed a new network architecture to solve the problem of poor robustness of semantic segmentation of skin lesion images. The main conclusions are as follows.

(1) We proposed a network framework with bidirectional feedback and dense connectivity. It experiments on two publicly available datasets, ISIC-2018 and PH2. And the results showed that our model has a high accuracy of 93.51% and 94.58%, respectively.
(2) Bi-directional feedback was applied in the model to establish close connections between codec networks, which can enhance feature reuse and information transfer. An edge module was added to the encoding to ensure that image edge information is not lost. Dense connections were used in the model to integrate semantic and contextual information at different resolutions.
(3) The segmentation performance of our network model was quantitatively compared with that of U-Net and other classical models. Experimental results showed that our network structure has better results than other network structures.

It is worth noting that although our method achieves an advantage in segmentation accuracy, it comes at the cost of running efficiency. In the future, researching a lightweight model with high accuracy and efficiency is the direction we need to work on.

## Data Availability

The original contributions presented in the study are included in the article/supplementary material, further inquiries can be directed to the corresponding authors.

## References

[B1] Abualigah L. Al-Okbi N. K. Abd Elaziz M. Houssein E. H. (2022). Boosting marine predators algorithm by salp swarm algorithm for multilevel thresholding image segmentation. Multimed. Tools Appl. 81, 16707–16742. 10.1007/s11042-022-12001-3 35261554 PMC8892122

[B2] Alom M. Z. Hasan M. Yakopcic C. Taha T. M. Asari V. K. (2018). Recurrent residual convolutional neural network based on U-Net (R2U-Net) for medical image segmentation. arXiv:1802.06955.

[B3] Badrinarayanan V. Kendall A. Cipolla R. (2017). SegNet: A deep convolutional encoder-decoder architecture for image segmentation. IEEE Trans. Patt. Anal. Mach. Intell. 39, 2481–2495. 10.1109/TPAMI.2016.2644615 28060704

[B4] Badshah N. Ahmad A. (2021). ResBCU-Net: Deep learning approach for segmentation of skin images. Biomed. Signal Process. Control 40, 103137–137. 10.1016/j.bspc.2021.103137

[B5] Cui R. Yang R. Liu F. Geng H. (2023). HD^2^A-Net: A novel dual gated attention network using comprehensive hybrid dilated convolutions for medical image segmentation. Comput. Biol. Med. 152, 106384. 10.1016/j.compbiomed.2022.106384 36493731

[B6] Da Silva R. R. Escarpinati M. C. Backes A. R. (2021). Sugarcane crop line detection from UAV images using genetic algorithm and Radon transform. Signal Image Video Process 15, 1723–1730. 10.1007/s11760-021-01908-3

[B7] Du X. F. Wang J. S. Sun W. Z. (2021). Densely connected U-Net retinal vessel segmentation algorithm based on multi-scale feature convolution extraction. Med. Phys. 48, 3827–3841. 10.1002/mp.14944 34028030

[B8] Garg S. Balkrishan J. (2022). Skin lesion segmentation in dermoscopy imagery. Int. Arab. J. Inf. Technol. 19, 29–37. 10.34028/iajit/19/1/4

[B9] Genitha C. H. Sowmya M. Sri T. (2020). Comparative analysis for the detection of marine vessels from satellite images using FCM and marker-controlled watershed segmentation algorithm. J. Indian Soc. Remote Sens. 48, 1207–1214. 10.1007/s12524-020-01148-x

[B10] Guo R. Shen X. J. Kang H. (2020). Image segmentation algorithm based on partial differential equation. J. Intell. Fuzzy Syst. 38, 3903–3909. 10.3233/jifs-179614

[B11] Indriyan T. Utoyo M. I. Rulaningtyas R. (2021). A new watershed algorithm for pothole image segmentation. Stud. Inf. Control 30, 131–139. 10.24846/v30i3y202112

[B12] Jia W. K. Zhang Z. H. Shao W. J. Ji Z. Hou S. J. (2022). RS-Net: Robust segmentation of green overlapped apples. Precis. Agric. 23, 492–513. 10.1007/s11119-021-09846-3

[B13] Lguensat R. Sun M. Fablet R. Mason E. Tandeo P. Chen G. (2018). “EddyNet: A deep neural network for pixel-wise classification of oceanic eddies,” in IEEE international geoscience and remote sensing symposium, 1764–1767. arXiv:1711.03954.

[B14] Li H. (2020). Examination on image segmentation method of ischemic optic neuropathy based on fuzzy clustering theory. J. Intell. Fuzzy Syst. 38, 3625–3633. 10.3233/JIFS-179585

[B15] Lin G. Milan A. Shen C. Reid I. (2017). “RefineNet: Multi-path refinement networks for high-resolution semantic segmentation,” in Proceedings of the IEEE conference on computer vision and pattern recognition, 5168–5177. arXiv:1611.06612.

[B16] Ma J. Chen J. Ng M. Huang R. Li Y. Li C. (2021). Loss odyssey in medical image segmentation. Med. Image Anal. 71, 102035. 10.1016/j.media.2021.102035 33813286

[B17] Qu Y. J. Wang Y. J. (2021). Segmentation of corpus callosum based on tensor fuzzy clustering algorithm. J. X-Ray Sci. Technol. 29, 931–944. 10.3233/XST-210928 34308897

[B18] Resma K. P. B. Nair M. S. (2021). Multilevel thresholding for image segmentation using krill herd optimization algorithm. J. King Saud. University-Comput. Inf. Sci. 33, 528–541. 10.1016/j.jksuci.2018.04.007

[B19] Ronneberger O. Fischer P. Brox T. (2015). “U-Net: Convolutional networks for biomedical image segmentation,” in International conference on medical image computing and computer-assisted intervention (Springer), 234–241. arXiv:1505.04597.

[B20] Rouhollahi A. Ilegbusi O. Foroosh H. (2020). Segmentation and pore structure estimation in SEM images of tissue engineering scaffolds using genetic algorithm. Ann. Biomed. Eng. 49, 1033–1045. 10.1007/s10439-020-02638-2 33057890

[B21] Roy A. G. Navab N. Wachinger C. (2018). “Concurrent spatial and channel ‘squeeze & excitation’ in fully convolutional networks,” in International conference on medical image computing and computer-assisted intervention, 421–429. arXiv:1803.02579.

[B22] Shelhamer E. Long J. Darrell T. (2017). Fully convolutional networks for semantic segmentation. IEEE Trans. Pattern Anal.Mach. Intell. 39, 640–651. 10.1109/TPAMI.2016.2572683 27244717

[B23] Singh S. Mittal N. Singh H. (2021). A multilevel thresholding algorithm using HDAFA for image segmentation. Soft Comput. 25, 10677–10708. 10.1007/s00500-021-05956-2

[B24] Sun K. Xiao B. Liu D. Wang J. D. (2019). “Deep high-resolution representation learning for human pose estimation,” in Proceedings of the IEEE/CVF conference on computer vision and pattern recognition, 693–5703. arXiv:1902.09212.

[B25] Tian C. Chen Y. G. (2020). Image segmentation and denoising algorithm based on partial differential equations. IEEE Sens. J. 20, 11935–11942. 10.1109/JSEN.2019.2959704

[B26] Tschandl P. Rosendahl C. Kittler H. (2018). The HAM10000 dataset, a large collection of multi-source dermatoscopic images of common pigmented skin lesions. Sci. data 5, 180161–180169. 10.1038/sdata.2018.161 30106392 PMC6091241

[B27] Tyagi A. Mehra R. (2020). An optimized CNN based intelligent prognostics model for disease prediction and classification from dermoscopy images. Multimed. Tools Appl. 79, 26817–26835. 10.1007/s11042-020-09074-3

[B28] Yang T. J. Zhou Y. D. Li L. Zhu C. H. (2020). DCU-Net: Multi-scale U-Net for brain tumor segmentation. J. X-Ray Sci. Technol. 28, 709–726. 10.3233/XST-200650 32444591

